# Individual and joint associations of anxiety disorder and depression with cardiovascular disease: A UK Biobank prospective cohort study

**DOI:** 10.1192/j.eurpsy.2023.2425

**Published:** 2023-07-05

**Authors:** Shinya Nakada, Frederick K. Ho, Carlos Celis-Morales, Caroline A. Jackson, Jill P. Pell

**Affiliations:** 1 School of Health and Wellbeing, University of Glasgow, Glasgow, UK; 2 School of Cardiovascular and Metabolic Health, University of Glasgow, Glasgow, UK; 3 Human Performance Laboratory, Education, Physical Activity and Health Research Unit, Universidad Católica del Maule, Talca, Chile; 4 Usher Institute, University of Edinburgh, Edinburgh, UK

**Keywords:** anxiety, cardiovascular, depression, myocardial, stroke

## Abstract

**Background:**

Growing evidence suggests that individuals with anxiety disorder have an elevated risk of cardiovascular disease (CVD) but few studies have assessed this association independently of or jointly with depression.

**Methods:**

We conducted a prospective cohort study using UK Biobank. Diagnoses of anxiety disorder, depression, and CVDs were ascertained through linked hospital admission and mortality data. Individual and joint associations between anxiety disorder and depression and CVD overall, as well as each of myocardial infarction, stroke/transient ischemic attack, and heart failure, were analyzed using Cox proportional hazard models and interaction tests.

**Results:**

Among the 431,973 participants, the risk of CVD was higher among those who had been diagnosed with anxiety disorder only (hazard ratio [HR] 1.72; 95% confidence interval [CI] 1.32–2.24), depression only (HR 2.07; 95% CI 1.79–2.40), and both conditions (HR 2.89; 95% CI 2.03–4.11) compared to those without these conditions, respectively. There was very little evidence of multiplicative or additive interaction. Results were similar for myocardial infarction, stroke/transient ischemic attack, and heart failure.

**Conclusions:**

Having anxiety is associated with the same magnitude of increased risk of CVD among people who do not have depression and those who do. Anxiety disorder should be considered for inclusion in CVD risk prediction and stratification, in addition to depression.

## Background

Mental health disorders and cardiovascular disease (CVD) affect more than 600 and 400 million people worldwide, respectively [1, 2]. One of the most common mental health disorders is anxiety disorder. Over the past 20 years, annual incident cases of anxiety disorder have increased globally by almost 50%, from 31 to 45 million [3]. Those with anxiety disorder are more likely to have CVD risk factors, such as hypertension and diabetes [4, 5]. A meta-analysis found a 40% excess risk of hypertension among those with anxiety disorder [4]. Further evidence has suggested an association between anxiety disorder and CVD. A meta-analysis reported 29 and 42% higher risk of incident CVD among women and men with anxiety disorder, respectively [6]. Single prospective cohort studies also showed associations between anxiety disorder and myocardial infarction (MI) (hazard ratio [HR] 2.51; 95% confidence interval [CI] 1.38–4.55) [7], and stroke (HR 1.14; 95% CI 1.03–1.25) [8] specifically.

However, these studies did not assess the association of anxiety disorder with CVDs independently of and jointly with depression. Existing evidence has shown both that depression is associated with CVD [9–11] and that, based on Mendelian randomization studies, the association is likely to be causal [12, 13]. Since anxiety disorder and depression often co-exist and may have shared or overlapping etiology [14–17], the mechanism by which they are associated with CVD may be common to both conditions.

To that end, our study aimed to examine the individual and joint associations of anxiety disorder and co-existing depression with a range of incident CVD (MI, stroke/transient ischemic attack [TIA], or heart failure) using the UK Biobank population cohort.

## Methods

### Study design and participants

We conducted a prospective cohort study using data from UK Biobank. UK Biobank recruited over 500,000 participants between 2007 and 2010 from the general population, aged 40 to 69 years. Participants visited one of the 22 assessment centers across England, Scotland, and Wales to provide their information and biological samples. We excluded participants who had experienced CVD, MI, stroke/TIA, or heart failure before the baseline assessment ascertained through either self-report or linkage to medical records and who were first admitted for anxiety disorder or depression after the baseline assessment.

### Measurements

The exposures of interest were anxiety disorder and depression diagnosed before the baseline assessment and were obtained through record linkage to hospital admission data: Health Episode Statistics (England and Wales) and Scottish Morbidity Records (Scotland). We defined anxiety disorder as F40–43 and depression as F32–33, using the International Classification of Diseases, 10th revision (ICD-10).

The outcomes of interest were incident (fatal or non-fatal) CVD, MI, stroke/TIA, and heart failure. The death certificate data were obtained from the National Health Service Information Centre (England and Wales) and the National Health Service Central Register Scotland (Scotland) and were available up to September 2021 in England and Wales, and October 2021 in Scotland. The hospital admission data were available up to September 2021 in England, July 2021 in Scotland, and February 2018 in Wales. Follow-up was censored at the date of relevant hospitalization or date of death, whichever occurred first. We defined CVD as an ICD-code of I20–25, I42.0, I42.6, I42.7, I42.9, I50, I60–64, and I110; MI as I21; stroke/TIA as I60–64 or G45, and heart failure as I42.0, I42.6, I42.7, I42.9, I50, or I110.

Covariates included sociodemographic (age, sex, ethnicity, and deprivation level) and lifestyle (alcohol intake, smoking status, sleep duration, TV viewing duration, physical activity, and body mass index [BMI]) factors. Age, sex, and ethnic group were self-reported by participants using a touchscreen questionnaire at baseline. Deprivation level was based on tertiles of the Townsend area deprivation index, which was derived from the postcode of residence using aggregated data on unemployment, car and home ownership, and household overcrowding [18]. Alcohol intake, smoking status, sleep, and TV viewing duration were self-reported using the touchscreen questionnaire at baseline. Physical activity was based on tertiles of metabolic equivalent minutes per week, which were derived from the self-completed, validated International Physical Activity Questionnaire [19]. BMI was calculated as weight/height^2^; height was measured to the nearest centimeter, using a Seca 202 stadiometer, and body weight was measured to the nearest 0.1 kg, using a Tanita BC-418 body composition analyzer by trained staff.

### Statistical analyses

Cox proportional hazard models were used to estimate the associations between anxiety disorder and depression and CVD outcomes, and the results expressed as HR and 95% CIs. Proportional hazard assumptions were checked by statistical tests based on Schoenfeld residuals. Separate models were run for each CVD outcome: overall CVD, MI, stroke/TIA, and heart failure. The main analyses comprised two stages. Firstly, the associations of each anxiety disorder and depression with CVD outcomes were analyzed by (1) univariable models, (2) multivariable models adjusted for sociodemographic confounders (age, sex, ethnic group, and deprivation level), and (3) multivariable models adjusted for both sociodemographic confounders and lifestyle factors (alcohol intake, smoking status, sleep duration, TV viewing duration, physical activity, and BMI). Because we could not establish the temporality between exposures and lifestyle factors, which could follow the exposure onset, we used models adjusted for sociodemographic confounders in the following analyses. Secondly, the individual and joint associations of anxiety disorder and depression with each of CVD, MI, stroke/TIA, and heart failure were determined, and multiplicative and additive interactions were tested. CVD outcomes were regressed on anxiety disorder, depression, their product term, and sociodemographic confounders. The HRs of anxiety disorders, depression, and their product term were used to estimate the individual and joint associations. The product term, and the relative excess risk due to interaction (RERI), were used to investigate the presence of multiplicative and additive interactions respectively. Missing values were < 5% and all analyses were complete case analyses.

Two different sensitivity analyses were performed to assess whether the additional use of self-reported exposures or primary care-diagnosed exposures and outcomes (available for 45% of the participants) affected the results. The case ascertainment in primary care settings was based on the UK Biobank Read codes mapping (https://biobank.ndph.ox.ac.uk/showcase/refer.cgi?id=592) (Supplementary Table S1). All analyses were conducted using R (version 3.5.3) with packages *survival* (version 3.2-7) and *interactionR* (version 0.1.5).

### Ethical considerations

UK Biobank was approved by the North-West Multi-Centre Research Ethics Committee (Ref: 11/NW/0382). The investigation conforms to the principles outlined in the Declaration of Helsinki. Informed consent was obtained from all individual participants included in the study. This work was conducted under the UK Biobank application number 7155.

## Results

**Figure 1.**
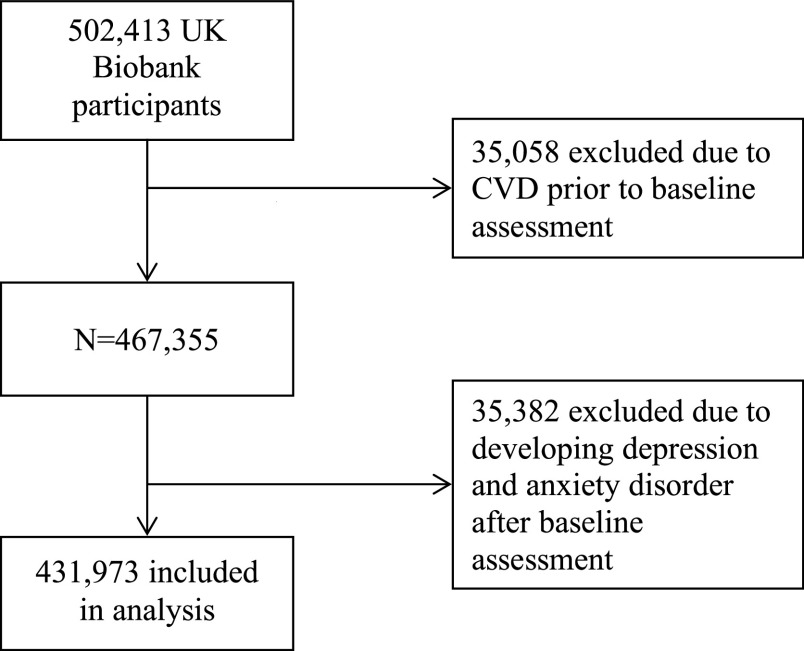
Flowchart of the participant selection process.

Of the 502,413 UK Biobank participants who had not withdrawn from the study and could be linked to hospital admission/death data, 35,058 were excluded because they had CVD before the baseline assessment and 35,382 were excluded because they developed depression or anxiety disorder after the baseline assessment. Consequently, 431,973 participants were included in the analyses (Figure 1).

Overall, 428,296 (99%) had no anxiety disorder or depression, 911 (0.2%) had anxiety disorder only, 2,427 (0.6%) had depression only and 399 (0.1%) had both anxiety disorder and depression (Table 1). Participants with either anxiety disorder or depression or both were more likely to be female and deprived than participants without either condition. They were also more likely to be a current smoker, inactive, and obese and to have longer sleep and TV viewing hours, but less likely to drink more alcohol than recommended. Those with both anxiety disorder and depression were more likely to have unhealthy lifestyles and develop CVDs than those with only anxiety disorder or depression (Table 1 and Supplementary Table S2).

**Table 1.** Characteristics of subjects by anxiety disorder and depression

	No anxiety disorder nor depression	Anxiety disorder only	Depression only	Anxiety disorder and depression
	*N* = 428,296	*N* = 911	*N* = 2,427	*N* = 339
Mean (SD) age (years)	56.1 (8.09)	55.8 (8.11)	55.0 (7.95)	53.7 (8.08)
Missing	11	0	0	0
Sex				
Female	235,675 (55.0%)	565 (62.0%)	1,482 (61.1%)	208 (61.4%)
Male	192,610 (45.0%)	346 (38.0%)	945 (38.9%)	131 (38.6%)
Missing	11	0	0	0
Ethnicity				
White	402,496 (94.5%)	855 (95.3%)	2,279 (94.8%)	320 (95.2%)
Non-White	23,496 (5.5%)	42 (4.7%)	126 (5.2%)	16 (4.8%)
Missing	2,304	14	22	3
Deprivation level				
Most deprived	141,972 (33.2%)	398 (43.9%)	1,265 (52.2%)	171 (50.7%)
Middle	142,814 (33.4%)	269 (29.7%)	627 (25.9%)	97 (28.8%)
Least deprived	142,967 (33.4%)	240 (26.5%)	531 (21.9%)	69 (20.5%)
Missing	543	4	4	2
Alcohol intake ≥14 units/week	161,619 (41.4%)	278 (34.2%)	721 (32.5%)	85 (28.3%)
Missing	37,522	97	207	339
Smoking status				
Never	240,855 (56.5%)	479 (53.3%)	1,064 (44.3%)	171 (51.0%)
Previous	143,039 (33.6%)	283 (31.5%)	785 (32.7%)	81 (24.2%)
Current	42,068 (9.88%)	137 (15.2%)	551 (23.0%)	83 (24.8%)
Missing	2,334	12	27	4
Mean (SD) sleep duration hours/day	7.15 (1.04)	7.17 (1.40)	7.23 (1.56)	7.33 (1.74)
Missing	3,132	21	54	8
Mean (SD) TV hours/day	2.72 (1.54)	3.08 (2.03)	3.32 (2.18)	3.55 (2.43)
Missing	7,609	30	85	10
Physical activity				
High	110,861 (33.4%)	208 (32.9%)	538 (30.4%)	80 (32.5%)
Middle	110,952 (33.4%)	186 (29.4%)	483 (27.3%)	66 (26.8%)
Low	110,602 (33.3%)	238 (37.7%)	748 (42.3%)	100 (40.7%)
Missing	95,881	279	658	93
Mean (SD) BMI (kg/m^2^)	27.2 (4.65)	27.6 (5.12)	28.9 (6.15)	28.8 (6.32)
Missing	2,376	11	37	5

Abbreviations: BMI, body mass index; *N*, number; SD, standard deviation; TV, television.

When adjusted for sociodemographic factors, both anxiety disorder and depression were associated with increased risk of CVD, MI, stroke/TIA, and heart failure (Table 2). Additional adjustments for lifestyle factors attenuated most of the estimates but anxiety disorder remained associated with overall CVD and stroke/TIA and depression remained associated with both of these and heart failure (Table 2).

**Table 2.** Associations of anxiety disorder and depression with cardiovascular disease, myocardial infarction, stroke/TIA, and heart failure

	Cardiovascular disease	Myocardial infarction	Stroke/TIA	Heart failure
	HR [95% CI]	HR [95% CI]	HR [95% CI]	HR [95% CI]
Model 1 (*n* = 431,973)				
Depression	1.92	1.59	1.73	2.34
	[1.68, 2.20]	[1.29, 1.97]	[1.44, 2.09]	[1.98, 2.78]
Anxiety disorder	1.76	1.59	1.72	1.62
	[1.43, 2.18]	[1.16, 2.18]	[1.31, 2.28]	[1.20, 2.18]
Model 2 (*n* = 429,091)				
Depression	2.16	1.79	1.96	2.69
	[1.88, 2.48]	[1.45, 2.22]	[1.62, 2.36]	[2.27, 3.19]
Anxiety disorder	2.00	1.81	1.91	1.80
	[1.62, 2.47]	[1.32, 2.48]	[1.44, 2.52]	[1.34, 2.44]
Model 3 (*n* = 302,373)				
Depression	1.83	1.17	1.97	2.16
	[1.54, 2.18]	[0.87, 1.58]	[1.57, 2.46]	[1.74, 2.70]
Anxiety disorder	1.61	1.38	1.56	1.47
	[1.21, 2.15]	[0.89, 2.15]	[1.06, 2.29]	[0.97, 2.24]

Abbreviations: CI, confidence interval; HR, hazard ratio; *n*, number; TIA, transient ischemic attack.

*Note*: Model 1: no adjustment. Model 2: adjusted for age, sex, ethnicity, and deprivation level. Model 3: adjusted for age, sex, ethnicity, deprivation level, alcohol intake, smoking status, sleep duration, television viewing, physical activity, and body mass index.

**Figure 2.**
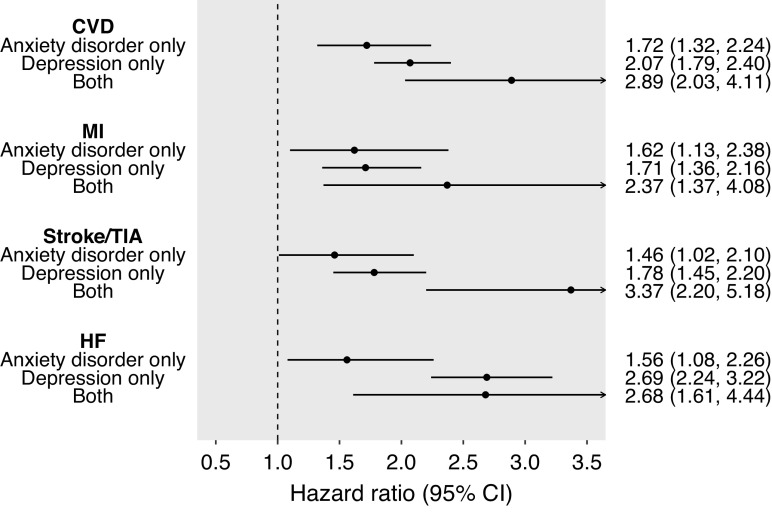
Individual and joint associations of anxiety disorder and depression with cardiovascular disease, myocardial infarction, stroke/TIA, and heart failure. CI, confidence interval; CVD, cardiovascular diseases; HF, heart failure; MI, myocardial infarction; TIA, transient ischemic attack. All models were adjusted for age, sex, ethnicity, and deprivation level.

Anxiety disorder was associated with CVD, MI, stroke/TIA, and heart failure independently of and jointly with depression when adjusted for sociodemographic factors (Table 3 and Figure 2). The risk of CVD was higher for those who had been diagnosed with anxiety disorder only (HR 1.72; 95% CI 1.32–2.24), depression only (HR 2.07; 95% CI 1.79–2.40), and both conditions (HR 2.89; 95% CI 2.03–4.11) referent to those without these conditions, respectively (Table 3). There was very little evidence of interaction on the multiplicative scale with the RERI values close to null (Table 3). We found similar results for MI, stroke/TIA, and heart failure.

**Table 3.** Joint associations and interactions of depression and anxiety disorder with cardiovascular disease, myocardial infarction, stroke/TIA, and heart failure

	No anxiety disorder HR [95% CI]	Anxiety disorder HR [95% CI]	Multiplicative interaction	RERI
Cardiovascular disease			0.81 [0.51, 1.29]	0.10 [−0.96, 1.41]
No depression	Reference	1.72 [1.32, 2.24]		
Depression	2.07 [1.78, 2.40]	2.89 [2.03, 4.11]		
Myocardial infarction			0.85 [0.42, 1.72]	0.03 [−1.30, 1.85]
No depression	Reference	1.62 [1.10, 2.38]		
Depression	1.71 [1.36, 2.16]	2.37 [1.37, 4.08]		
Stroke/TIA			1.29 [0.71, 2.35]	1.13 [−0.27, 3.01]
No depression	Reference	1.46 [1.01, 2.1]		
Depression	1.78 [1.45, 2.20]	3.37 [2.20, 5.18]		
Heart failure			0.64 [0.33, 1.22]	−0.58 [−1.95, 1.30]
No depression	Reference	1.56 [1.08, 2.26]		
Depression	2.69 [2.24, 3.22]	2.68 [1.61, 4.44]		

Abbreviations: CI, confidence interval; HR, hazard ratio; RERI, relative risk due to interaction; TIA, transient ischemic attack.

*Note*: All models were adjusted for anxiety disorder, depression, anxiety disorder × depression, age, sex, ethnicity, and deprivation level.

In the sensitivity analysis, the addition of self-reported anxiety disorder and depression attenuated overall associations but there were individual and joint associations of both conditions with overall CVD (Supplementary Table S3). Similarly, the addition of primary care data showed individual and joint associations with MI (Supplementary Table S4).

## Discussion

### Primary findings

Our study examined the individual and joint associations of anxiety disorder and depression with incident CVD and its subtypes (MI, stroke/TIA, and heart failure) using the UK Biobank population cohort. Anxiety disorder and depression were associated with incident CVD independently of each other and their co-existence was associated with a higher risk than either condition in isolation. However, there was no evidence of interaction implying that the sum of their individual associations with CVD was almost identical to their joint association based on RERI. Therefore, having anxiety contributes to the same relative excess risk of CVD regardless of whether depression is also present.

### Comparison with the literature

Our findings would be consistent with different mechanisms by which anxiety disorder and depression potentially increase CVD risk. This would be biologically plausible based on previous studies suggesting different brain structures between these conditions [20–22]. For example, a recent meta-analysis found lower gray matter volume in the superior temporal gyrus of people with anxiety disorder, compared with those with depression. This structural difference could affect reactions to stimuli, which could lead to distinctive behaviors associated with anxiety disorder [20].

Our findings are consistent with previous evidence that has suggested similar individual and joint associations of anxiety disorder and depression with CVD risks [23, 24] but a few studies reported conflicting findings. A US prospective cohort study did not find an association between anxiety disorder and CVD, ascertained through self-reported symptoms, independent of depression (HR 1.06; 95% CI 0.80–1.39) [25]. Their conflicting results may be explained by reporting bias or the inclusion of milder cases of anxiety disorder. Similarly, a German prospective cohort study did not show individual or joint associations for depression and sub-types of anxiety disorder (e.g., generalized anxiety disorder) [26]. This finding might be explained by sub-types of anxiety disorders operating differently from composite anxiety disorder and highlights the need for future research powered to investigate sub-group differences.

### Limitations

There are some limitations to our study. First, ascertainment of anxiety disorder and depression using hospital admission may be incomplete and less likely to include people with less severe conditions since they are often treated in primary care settings [27]. However, we investigated how the additional use of self-reports or primary care data affected our findings in sensitivity analyses and still found individual and joint associations with CVD and MI. Second, we only ascertained whether anxiety disorder and depression had ever occurred over a time period. We could not record varying anxiety severity or remitting–relapsing fluctuations, and therefore could not investigate the impact these may have on the relationship between anxiety and CVD [28]. Third, our findings may not be generalizable to the whole UK population because UK Biobank participants are more likely to be white, affluent, and healthy than the national survey data [29].

### Implications

Our findings suggest that anxiety disorder and depression may increase the risk of CVD independently of each other. As we are facing an increasing number of people living with anxiety disorder, their higher risk of CVD should be noted. In recent years, some CVD risk prediction algorithms (e.g., QRISK3) have included depression [30]. Given the findings of our study, the added value of including anxiety disorder in risk prediction and stratification should be explored.

## Conclusions

We examined the individual and joint associations of anxiety disorder and depression with incident CVD, MI, stroke/TIA, and heart failure among the middle- and old-aged UK population using the linkage of UK Biobank to hospital admission and mortality data. We found a higher risk of CVD among those with anxiety disorder only and depression only and the highest risk among those with both conditions, but no evidence of interaction on multiplicative or additive scales. This implies that the sum of their individual associations with CVD was almost identical to their joint association and therefore having anxiety contributes the same relative excess risk of CVD regardless of the presence of depression. One explanation is the existence of different mechanisms by which anxiety disorder and depression increase the CVD risk and this requires further research. Meanwhile, as has happened with depression, anxiety should be considered for inclusion in CVD risk prediction and stratification.

## Data Availability

The data that support the findings of this study are available from the UK Biobank, but restrictions apply to the availability of these data, which were used under license for the current study, and so are not publicly available. Data are, however, available from the authors upon reasonable request and with permission from the UK Biobank.

## Supplementary material

For supplementary material accompanying this paper visit http://doi.org/10.1192/j.eurpsy.2023.2425. click here to view supplementary material

## References

[1] GBD 2019 Mental Disorders Collaborators. Global, regional, and national burden of 12 mental disorders in 204 countries and territories, 1990–2019: a systematic analysis for the Global Burden of Disease Study 2019. Lancet Psychiatry. 2022;9(2):137–50. 35026139 10.1016/S2215-0366(21)00395-3 PMC8776563

[2] Roth GA, Johnson C, Abajobir A, Abd-Allah F, Abera SF, Abyu G, et al. Global, regional, and national burden of cardiovascular diseases for 10 causes, 1990 to 2015. J Am Coll Cardiol. 2017;70(1):1–25. 28527533 10.1016/j.jacc.2017.04.052 PMC5491406

[3] Xiong P, Liu M, Liu B, Hall BJ. Trends in the incidence and DALYs of anxiety disorders at the global, regional, and national levels: estimates from the Global Burden of Disease Study 2019. J Affect Disord. 2022;297:83–93. 34678404 10.1016/j.jad.2021.10.022

[4] Lim LF, Solmi M, Cortese S. Association between anxiety and hypertension in adults: a systematic review and meta-analysis. Neurosci Biobehav Rev. 2021;131:96–119. 34481847 10.1016/j.neubiorev.2021.08.031

[5] Mersha AG, Tollosa DN, Bagade T, Eftekhari P. A bidirectional relationship between diabetes mellitus and anxiety: a systematic review and meta-analysis. J Psychosom Res. 2022;162:110991. 36081182 10.1016/j.jpsychores.2022.110991

[6] Smaardijk VR, Lodder P, Kop WJ, van Gennep B, Maas A, Mommersteeg PMC. Sex- and gender-stratified risks of psychological factors for incident ischemic heart disease: systematic review and meta-analysis. J Am Heart Assoc. 2019;8(9):e010859. 31030598 10.1161/JAHA.118.010859 PMC6512085

[7] Janszky I, Ahnve S, Lundberg I, Hemmingsson T. Early-onset depression, anxiety, and risk of subsequent coronary heart disease: 37-year follow-up of 49,321 young Swedish men. J Am Coll Cardiol. 2010;56(1):31–7. 20620714 10.1016/j.jacc.2010.03.033

[8] Lambiase MJ, Kubzansky LD, Thurston RC. Prospective study of anxiety and incident stroke. Stroke. 2014;45(2):438–43. 24357656 10.1161/STROKEAHA.113.003741 PMC4354776

[9] Van der Kooy K, van Hout H, Marwijk H, Marten H, Stehouwer C, Beekman A. Depression and the risk for cardiovascular diseases: systematic review and meta analysis. Int J Geriatr Psychiatry. 2007;22(7):613–26. 17236251 10.1002/gps.1723

[10] Harshfield EL, Pennells L, Schwartz JE, Willeit P, Kaptoge S, Bell S, et al. Association between depressive symptoms and incident cardiovascular diseases. JAMA. 2020;324(23):2396–405. 33320224 10.1001/jama.2020.23068 PMC7739139

[11] Wei J, Hou R, Zhang X, Xu H, Xie L, Chandrasekar EK, et al. The association of late-life depression with all-cause and cardiovascular mortality among community-dwelling older adults: systematic review and meta-analysis. Br J Psychiatry. 2019;215(2):449–55. 30968781 10.1192/bjp.2019.74

[12] Li GH, Cheung CL, Chung AK, Cheung BM, Wong IC, Fok MLY, et al. Evaluation of bi-directional causal association between depression and cardiovascular diseases: a Mendelian randomization study. Psychol Med. 2022;52(9):1765–76. 33032663 10.1017/S0033291720003566

[13] Tang B, Yuan S, Xiong Y, He Q, Larsson SC. Major depressive disorder and cardiometabolic diseases: a bidirectional Mendelian randomisation study. Diabetologia. 2020;63(7):1305–11. 32270255 10.1007/s00125-020-05131-6 PMC7286869

[14] Lamers F, van Oppen P, Comijs HC, Smit JH, Spinhoven P, van Balkom AJ, et al. Comorbidity patterns of anxiety and depressive disorders in a large cohort study: the Netherlands Study of Depression and Anxiety (NESDA). J Clin Psychiatry. 2011;72(3):3397. 10.4088/JCP.10m06176blu 21294994

[15] Etkin A, Schatzberg AF. Common abnormalities and disorder-specific compensation during implicit regulation of emotional processing in generalized anxiety and major depressive disorders. Am J Psychiatry. 2011;168(9):968–78. 21632648 10.1176/appi.ajp.2011.10091290

[16] Goodkind M, Eickhoff SB, Oathes DJ, Jiang Y, Chang A, Jones-Hagata LB, et al. Identification of a common neurobiological substrate for mental illness. JAMA Psychiatry. 2015;72(4):305–15. 25651064 10.1001/jamapsychiatry.2014.2206 PMC4791058

[17] McTeague LM, Huemer J, Carreon DM, Jiang Y, Eickhoff SB, Etkin A. Identification of common neural circuit disruptions in cognitive control across psychiatric disorders. Am J Psychiatry. 2017;174(7):676–85. 28320224 10.1176/appi.ajp.2017.16040400 PMC5543416

[18] Townsend P, Phillimore P, Beattie A. Health and deprivation: inequality and the North. Oxfordshire: Routledge; 1988.

[19] Craig CL, Marshall AL, Sjöström M, Bauman AE, Booth ML, Ainsworth BE, et al. International physical activity questionnaire: 12-country reliability and validity. Med Sci Sports Exerc. 2003;35(8):1381–95. 12900694 10.1249/01.MSS.0000078924.61453.FB

[20] Serra-Blasco M, Radua J, Soriano-Mas C, Gómez-Benlloch A, Porta-Casteràs D, Carulla-Roig M, et al. Structural brain correlates in major depression, anxiety disorders and post-traumatic stress disorder: a voxel-based morphometry meta-analysis. Neurosci Biobehav Rev. 2021;129:269–81. 34256069 10.1016/j.neubiorev.2021.07.002

[21] van Tol M-J, van der Wee NJ, van den Heuvel OA, Nielen MM, Demenescu LR, Aleman A, et al. Regional brain volume in depression and anxiety disorders. Arch Gen Psychiatry. 2010;67(10):1002–11. 20921116 10.1001/archgenpsychiatry.2010.121

[22] Lai CH, Wu YT. The gray matter alterations in major depressive disorder and panic disorder: putative differences in the pathogenesis. J Affect Disord. 2015;186:1–6. 26208214 10.1016/j.jad.2015.07.022

[23] Walters K, Rait G, Petersen I, Williams R, Nazareth I. Panic disorder and risk of new onset coronary heart disease, acute myocardial infarction, and cardiac mortality: cohort study using the general practice research database. Eur Heart J. 2008;29(24):2981–8. 18948354 10.1093/eurheartj/ehn477

[24] Seldenrijk A, Vogelzangs N, Batelaan NM, Wieman I, van Schaik DJ, Penninx BJ. Depression, anxiety and 6-year risk of cardiovascular disease. J Psychosom Res. 2015;78(2):123–9. 25454680 10.1016/j.jpsychores.2014.10.007

[25] Karlsen HR, Saksvik-Lehouillier I, Stone KL, Schernhammer E, Yaffe K, Langvik E. Anxiety as a risk factor for cardiovascular disease independent of depression: a prospective examination of community-dwelling men (the MrOS study). Psychol Health. 2021;36(2):148–63. 32584189 10.1080/08870446.2020.1779273 PMC7759580

[26] Tully PJ, Baune BT. Comorbid anxiety disorders alter the association between cardiovascular diseases and depression: the German National Health Interview and Examination Survey. Soc Psychiatry Psychiatr Epidemiol. 2014;49(5):683–91. 24166703 10.1007/s00127-013-0784-x

[27] Ansseau M, Dierick M, Buntinkx F, Cnockaert P, De Smedt J, Van Den Haute M, et al. High prevalence of mental disorders in primary care. J Affect Disord. 2004;78(1):49–55. 14672796 10.1016/s0165-0327(02)00219-7

[28] Peter RS, Meyer ML, Mons U, Schöttker B, Keller F, Schmucker R, et al. Long-term trajectories of anxiety and depression in patients with stable coronary heart disease and risk of subsequent cardiovascular events. Depress Anxiety. 2020;37(8):784–92. 32237189 10.1002/da.23011

[29] Fry A, Littlejohns TJ, Sudlow C, Doherty N, Adamska L, Sprosen T, et al. Comparison of sociodemographic and health-related characteristics of UK Biobank participants with those of the general population. Am J Epidemiol. 2017;186(9):1026–34. 28641372 10.1093/aje/kwx246 PMC5860371

[30] Hippisley-Cox J, Coupland C, Brindle P. Development and validation of QRISK3 risk prediction algorithms to estimate future risk of cardiovascular disease: prospective cohort study. BMJ. 2017;357:j2099. 28536104 10.1136/bmj.j2099 PMC5441081

