# Supplementary material for: Individual and joint associations of anxiety disorder and depression with cardiovascular disease: A UK Biobank prospective cohort study
Source: Eur Psychiatry. 2023 Jul 5;66(1):e54. doi: 10.1192/j.eurpsy.2023.2425 (PMC10377450; doi:10.1192/j.eurpsy.2023.2425)
Supplement: Supplementary file 1 [file S0924933823024252sup001.docx]

**Supplementary Materials for**

**Individual and joint associations of anxiety disorder and depression with cardiovascular disease: A UK Biobank prospective cohort study**

Supplementary Table 1. READ codes to ascertain depression and anxiety disorder

Supplementary Table 2. Outcome summary by anxiety disorder and depression

Supplementary Table 3. Individual and joint associations of self-reported/hospitalised depression and anxiety disorder with cardiovascular disease, myocardial infarction, stroke/TIA, and heart failure

Supplementary Table 4. Individual and joint associations of depression and anxiety disorder with cardiovascular disease, myocardial infarction, stroke/TIA, and heart failure among participants with primary care data

Supplementary Table 1. READ codes to ascertain depression and anxiety disorder

|  | READ2 | READ3 |
| --- | --- | --- |
| Depression | Eu32., Eu320, Eu321, Eu322, Eu323, Eu324, Eu325, Eu326, Eu327, Eu328, Eu329, Eu32A, Eu32B, Eu32y, Eu32z, Eu33., Eu330, Eu331, Eu332, Eu333, Eu334, Eu33y, Eu33z | 2257., E0043, E1121, E1122, E1123, E1124, E1126, E1131, E1132, E1133, E1134, E1135, E1136, E1137, E11y2, E130., E2B0., E2B1., Eu320, Eu321, Eu322, Eu323, Eu32y, Eu32z, Eu330, Eu331, Eu332, Eu333, Eu334, Eu33y, Eu33z, X00SO, X00SQ, X00SR, X00SS, X00SU, XE1Y0, XE1Y1, XE1YC, XE1ZY, XE1ZZ, XE1Za, XE1Zb, XE1Zc, XE1Zd, XE1Ze, XE1Zf, XM1GC, XSEGJ, XSGok, XSGol, XSGom, XSGon, Xa0wV, XaB9J, XaCHo, XaCHr, XaCHs, XaCIs, XaCIt, XaCIu, XaX53, XaX54, XaY2C |
| Anxiety disorder | Eu4.., Eu40., Eu400, Eu401, Eu402, Eu403, Eu40y, Eu40z, Eu41., Eu410, Eu411, Eu412, Eu413, Eu41y, Eu41z, Eu42., Eu420, Eu421, Eu422, Eu42y, Eu42z, Eu43., Eu430, Eu431, Eu432, Eu433, Eu434, Eu435, Eu43y, Eu43z | 1B1L., 1BE.., E200., E2002, E2004, E2021, E2023, E2024, E2025, E2027, E2028, E2029, E202E, E203., E2030, E2031, E2753, E280., E281., E282., E2830, E2831, E284., E29.., E290., E291., E2924, E2925, E292y, E293., E2930, E2931, E2932, E294., E29y2, E29y4, Eu40., Eu400, Eu402, Eu40y, Eu40z, Eu41., Eu410, Eu413, Eu41y, Eu41z, Eu420, Eu421, Eu422, Eu42y, Eu42z, Eu430, Eu432, Eu43y, Eu43z, Ua18L, Ua18k, Ua1qS, Ua1qU, Ua1qV, Ua1qW, Ua1qX, Ua1qY, Ua1qa, Ua1qc, Ua1qd, Ua1qe, Ua1qf, Ua1qg, Ua1qh, Ua1qi, Ua1qj, Ua1qk, Ua1ql, Ua1qm, Ua1qn, Ua1qo, Ua1qp, Ua1qs, Ua1qt, X00SV, X00SW, X00SX, X00SY, X00SZ, X00Sa, X00Sb, X00Sc, X00Sd, X00Se, X00Sf, X00Sl, X00Sr, X50G3, X50GI, X75YV, X761d, X761n, X761q, X761t, X761u, X761y, X7627, X7628, X7629, X762A, X762C, X762E, X762F, X762G, X762H, X762T, X762c, X762d, X762e, X762f, X762g, X762h, X762i, X762j, X762l, X762m, X762n, X762p, X762q, X764L, X764N, X764O, X764P, X764Q, X78wp, XE0rb, XE1Y7, XE1YA, XE1Ym, XE1Yo, XE1Yp, XE1Zj, XM001, XM0Ak, XM0As, XM0At, XM1Q3, Xa00r, Xa00s, Xa02G, Xa02H, Xa02I, Xa03z, Xa18j, Xa18v, Xa1Ev, Xa1a8, Xa2kf, Xa3Vj, Xa3Vk, Xa3Vl, Xa3WH, Xa3WI, Xa3WJ, Xa7k9, Xa7kB, XaC2u, XaIo7, XaKVA, XaX55, XaX56, XaX58 |

Supplementary Table 2. Outcome summary by anxiety disorder and depression

|  | **No anxiety disorder nor depression** | **Anxiety disorder only** | **Depression only** | **Anxiety disorder and depression** | **Total** |
| --- | --- | --- | --- | --- | --- |
|  | **N (%)** | **N (%)** | **N (%)** | **N (%)** | **N (%)** |
|  | 42,8296 (99.1) | 911 (0.2) | 2,427 (0.6) | 339 (0.08) |  |
| CVD | 17,205 (4.02) | 56 (6.15) | 177 (7.29) | 31 (9.14) | 17,469 (4.0) |
| MI | 8,465 (1.98) | 26 (2.85) | 73 (3.01) | 13 (3.83) | 8,577 (2.0) |
| Stroke/TIA | 10,077 (2.35) | 29 (3.18) | 90 (3.71) | 21 (6.19) | 10,217 (2.4) |
| HF | 9,167 (2.14) | 28 (3.07) | 120 (4.94) | 15 (4.42) | 9,330 (2.2) |

N, number; CVD, cardiovascular diseases; MI, myocardial infarction; TIA, transient ischaemic attack; HF, heart failure.

All models were adjusted for age, sex, ethnicity, and deprivation level.

Supplementary Table 3. Individual and joint associations of self-reported/hospitalised depression and anxiety disorder with cardiovascular disease, myocardial infarction, stroke/TIA, and heart failure

|  | No anxiety disorder  HR [95% CI] | Anxiety disorder  HR [95% CI] | Multiplicative interaction | RERI |
| --- | --- | --- | --- | --- |
| **Cardiovascular disease** |  |  | 1.01 [0.77, 1.32] | 0.04 [-0.30, 0.42] |
| No depression | Reference | 1.22 [1.07, 1.40] |  |  |
| Depression | 1.12 [1.04, 1.22] | 1.38 [1.11, 1.73] |  |  |
| **Myocardial infaction** |  |  | 1.16 [0.78, 1.72] | 0.18 [-0.28, 0.74] |
| No depression | Reference | 1.13 [0.93, 1.38] |  |  |
| Depression | 1.01 [0.90, 1.14] | 1.33 [0.96, 1.83] |  |  |
| **Stroke/TIA** |  |  | 1.16 [0.81, 1.66] | 0.19 [-0.23, 0.70] |
| No depression | Reference | 1.12 [0.93, 1.34] |  |  |
| Depression | 1.04 [0.94, 1.16] | 1.35 [1.01, 1.81] |  |  |
| **Heart failure** |  |  | 1.09 [0.75, 1.57] | 0.15 [-0.32, 0.72] |
| No depression | Reference | 1.13 [0.93, 1.37] |  |  |
| Depression | 1.24 [1.11, 1.38] | 1.52 [1.13, 2.04] |  |  |

HR, hazard ratio; CI, confidence interval; RERI, relative risk due to interaction; TIA, transient ischaemic attack.

All models were adjusted for anxiety disorder, depression, anxiety disorder*depression, age, sex, ethnicity, and deprivation level.

Supplementary Tables 4. Individual and joint associations of depression and anxiety disorder with cardiovascular disease, myocardial infarction, stroke/TIA, and heart failure among participants with primary care data

|  | No anxiety disorder  HR [95% CI] | Anxiety disorder  HR [95% CI] | Multiplicative interaction | RERI |
| --- | --- | --- | --- | --- |
| **Cardiovascular disease** |  |  | 1.06 [0.90, 1.26] | 0.12 [-0.11, 0.36] |
| No depression | Reference | 1.09 [0.99, 1.20] |  |  |
| Depression | 1.35 [1.24, 1.47] | 1.56 [1.39, 1.76] |  |  |
| **Myocardial infaction** |  |  | 1.0 [0.79, 1.28] | 0.07 [-0.27, 0.42] |
| No depression | Reference | 1.18 [1.04, 1.35] |  |  |
| Depression | 1.34 [1.19, 1.52] | 1.59 [1.34, 1.89] |  |  |
| **Stroke/TIA** |  |  | 1.03 [0.84, 1.25] | 0.07 [-0.19, 0.35] |
| No depression | Reference | 1.09 [0.98, 1.22] |  |  |
| Depression | 1.36 [1.23, 1.50] | 1.53 [1.33, 1.75] |  |  |
| **Heart failure** |  |  | 1.06 [0.83, 1.34] | 0.11 [-0.22, 0.45] |
| No depression | Reference | 1.06 [0.93, 1.21] |  |  |
| Depression | 1.43 [1.27, 1.60] | 1.60 [1.36, 1.88] |  |  |

HR, hazard ratio; CI, confidence interval; RERI, relative risk due to interaction; TIA, transient ischaemic attack.

All models were adjusted for anxiety disorder, depression, anxiety disorder*depression, age, sex, ethnicity, and deprivation level.
